# *OsSPLs* Regulate Male Fertility in Response to Different Temperatures by Flavonoid Biosynthesis and Tapetum PCD in PTGMS Rice

**DOI:** 10.3390/ijms23073744

**Published:** 2022-03-29

**Authors:** Yujun Sun, Ming Fu, Lei Wang, Yunxiu Bai, Xueliang Fang, Qian Wang, Ying He, Hanlai Zeng

**Affiliations:** MOA Key Laboratory of Crop Ecophysiology and Farming System in the Middle Reaches of the Yangtze River, College of Plant Science and Technology, Huazhong Agricultural University, Wuhan 430070, China; yujunsun@webmail.hzau.edu.cn (Y.S.); fuming2016@webmail.hzau.edu.cn (M.F.); wangleistudent@webmail.hzau.edu.cn (L.W.); byx@webmail.hzau.edu.cn (Y.B.); fxl1995@webmail.hzau.edu.cn (X.F.); samwang@webmail.hzau.edu.cn (Q.W.)

**Keywords:** *OsSPL*, PTGMS, pollen sterility, temperature change, the flavonoid pathway, tapetum PCD

## Abstract

Photoperiod and thermo-sensitive genic male sterile (PTGMS) rice is an important resource for two line hybrid rice production. The SQUAMOSA–promoter binding, such as the (*SPL*) gene family, encode the plant specific transcription factors that regulate development and defense responses in plants. However, the reports about *SPLs* participating in male fertility regulation are limited. Here, we identified 19 *OsSPL* family members and investigated their involvement in the fertility regulation of the PTGMS rice lines, PA2364S and PA2864S, with different fertility transition temperatures. The results demonstrated that *OsSPL2*, *OsSPL4*, *OsSPL16* and *OsSPL17* affect male fertility in response to temperature changes through the MiR156-*SPL* module. WGCNA (weighted gene co-expression network analysis) revealed that *CHI* and *APX1* were co-expressed with *OsSPL17*. Targeted metabolite and flavonoid biosynthetic gene expression analysis revealed that *OsSPL17* regulates the expression of flavonoid biosynthesis genes *CHI,* and the up regulation of flavanones (eriodictvol and naringenin) and flavones (apigenin and luteolin) content contributed to plant fertility. Meanwhile, *OsSPL17* negatively regulates *APX1* to affect APX (ascorbate peroxidase) activity, thereby regulating ROS (reactive oxygen species) content in the tapetum, controlling the PCD (programmed cell death) process and regulating male fertility in rice. Overall, this report highlights the potential role of *OsSPL* for the regulation of male fertility in rice and provides a new insight for the further understanding of fertility molecular mechanisms in PTGMS rice.

## 1. Introduction

Precise coordination of gene expression is essential during plant growth and development, and transcriptional regulation is an important mechanism for controlling gene expression. Transcription factors (TFs) regulate gene expression on the transcriptional level and control various plant life processes by interacting with proteins. Currently, most known TFs have been classified according to specific DNA-binding structural domains and conserved motifs as family classification criteria, and these structural domains are relatively conserved in terms of family evolution [1]. The SQUAMOSA-promoter binding, such as the (*SPL*) genes, encode a class of plant specific TFs, and this family contains a highly conserved DNA-binding structural domain consisting of 76 amino acids called the SBP (SQUA promoter-binding proteins) domain. The *SPL* family plays an important role in the gene network that regulates plant development and defense responses [2,3].

*AmSBP1*/*P2* were identified from *Antirrhinum majus* and these two genes could control flower development by binding to the promoter of the *SQUAMOSA* gene [4]. To investigate the role of *SBP-box* genes in plant growth and development, researchers searched for their homologs in the model plant *Arabidopsis thaliana* at first, and sixteen *AtSPL* have been identified [5,6]. The proteins encoded by these genes share a sequence region of high similarity, which is the SBP structural domain encoded by the SBP-box. Commonly, the *SPL* family varies greatly between species. Currently, *SPL* has been known to regulate a variety of important biological processes, including plant microspore development [7], male fertility [8], nutrient organ development [9], flower and fruit development [10], hormone signaling [11] and abiotic stress regulation [12].

MircoRNA are endogenous noncoding small RNAs with 21–24 nucleotides that bind to complementary mRNAs to repress the translation process, regulate gene expression and reduce protein expression levels [13]. Furthermore, miR156 is a relatively conserved miRNA in the plant kingdom, which eleven *OsSPL* were found to be targeted by, and the miR156/*SPL* module plays an important role in plant tissue development [14,15]. In terms of rice agronomic traits and yield contribution, *OsSPL2, OsSPL16*, *OsSPL17* and *OsSPL18* were involved in controlling plant height and tiller number at the tillering stage, while overexpression of miR156 significantly increased the number of tillers and decreased the number of grains per spike by regulating the targets *OsSPL2*, *OsSPL12*, *OsSPL13* and *OsSPL14* [15]. However, high expression levels of *OsSPL14* at the reproductive stage promote spike branching and obtain higher seed yield in rice [16,17]. In *OsSPL18* knockout rice mutants, a significant decrease in grain width, thickness, spikelet length and the number of grains was found, and in contrast an increase in tiller number. Subsequently, the miR156k-*OsSPL18d* regulatory module was found to regulate rice grain number by affecting cell proliferation to regulate spikelet shell development [18]. The miR156-*OsSPL3*/*12* module was found to directly activate *OsMADS50* to regulate crown root development in rice [19]. The role of the miR156/*OsSPL* module in plant stress responses has attracted the attention of researchers. Under adverse conditions of biotic stress, the plant immune system is an important component of balanced growth and development. It has been shown that the miR156/*SPL9* module generates resistance to *Pseudomonas syringae*, by regulating the expression of the defense gene *FLS2*. However, *SPL9* binds to specific motifs within the miR528 promoter region to activate miR528 expression and regulate L-Ascorbate Oxidase (AO) levels to generate a defense response to rice stripe virus (RSV) [20]. In response to abiotic stress, miR156 has been shown to target *SPL* in Arabidopsis in response to heat stress. The expression of miR156 increases at high temperatures and *SPL2/9/11* gene expression decreases, to counteract the negative effects of heat stress on plant growth [21].

Rice (*Oryza sativa* L.) is a staple food for nearly half of the world’s population and is one of the most important food crops [22]. To cope with the food requirements of the rapid growth of the world population, efficient breeding to improve crop productivity is essential [23]. Two line hybrid rice uses photoperiod- and thermo-sensitive genic male sterile (PTGMS) lines to produce hybrid seeds, thus eliminating the limitations of the restorer–maintainer relationship of three-line hybrids system, and achieving higher efficiency in excellent hybrid configurations and hybrid seed production [24]. Male fertility in PTGMS rice is controlled by temperature and light conditions, but the mechanism of fertility regulation is unclear. Recent research found that *OsSPL* is essential for meiosis with rice and plays a regulatory role in callus deposition, tapetum development and anther wall differentiation [25].

Although members of the *SPL* gene family have been relatively investigated in plant development and stress response, whether the *SPL* gene family, or certain of its members, regulates male fertility in PTGMS rice is still unclear. In this study, we performed a genomewide identification of the *SPL* gene family members in rice and analyzed phylogenetic relationships and collinearity in the genome. Expression analysis of the near isogenic lines of material PA2364S and PA2864S with different fertility transformation temperatures was performed. The relationship between *OsSPL* and male fertility at the same temperature and the effect of different temperatures induction of *OsSPL* on the level of fertility were investigated. The potential regulatory pathway of *OsOPL* was explored using protein structure analysis and WGCNA (weighted gene co-expression network analysis). Finally, a flavonoid metabolism and TUNEL (transferase mediated dUTP nick end labeling) assay were used to verify that *OsOPL17* might be involved in regulating PTGMS rice fertility by affecting the biosynthetic process of the flavonoid and tapetum PCD (programmed cell death) process. The findings provide a new insight to resolve the mechanism of fertility in PTGMS rice.

## 2. Results

### 2.1. Abnormal Development of PA64S Pollen Grains Producing Male Sterile Plants under High Temperature

**Figure 1:**
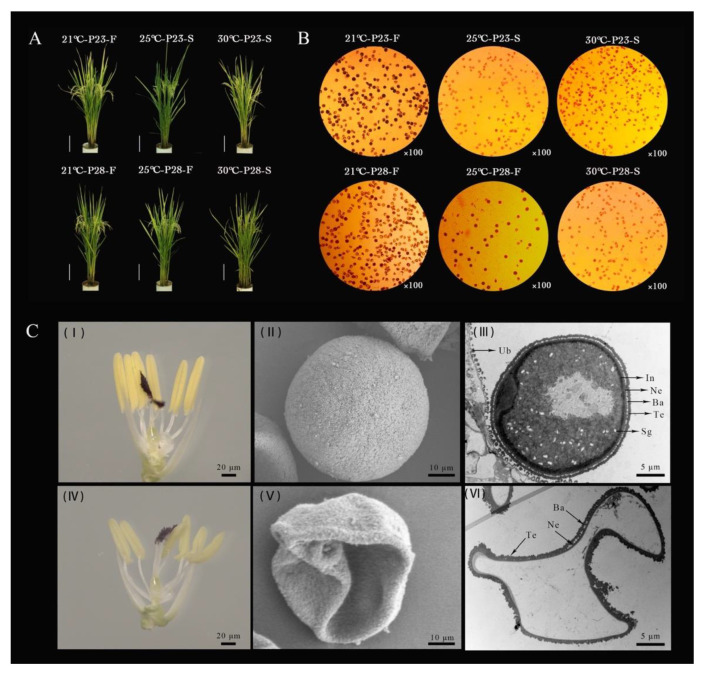
Characteristics of fertile and sterile plants and pollen phenotypes of PA64S. (**A**) The phenotype of PA2364S and PA2864S plants under different temperature treatments. (**B**) Pollen fertility of PA2364S and PA2864S under different temperature treatments. (**C**) Anther phenotypes and electron microscopic observation of pollen grains in fertile and sterile plants. (**I**–**III**) show anther and SEM and TEM of pollen in fertile plants, respectively. (**IV**–**VI**) show anther and SEM and TEM of pollen in plants, respectively. SG, starch granule; In, intine; Ne, nexine; Ba, bacula; Te, texine; Ub, Ubisch body. 23 °C-P23-F. Fertile plants under 23 °C treatment in PA2364S.

First, the temperature experimental system for pollen fertility transformations was built using one pair of PTGMS rice lines, PA2364S and PA2864S, with different critical temperatures. We obtained fertile and sterile material for PA2364S and PA2864S, which were treated by three temperatures, respectively. Differences in male fertility and seed setting were observed between PA2364S and PA2864S after the 25 °C treatment (Figure 1A,B). The iodine staining and setting rates were zero after the 30 °C treatment, and approximately 39.30% and 37.35% for PA2364S and PA2864S, respectively, after the 21 °C treatment (Figure 1B and Table 1). Microscopic observation of the anthers and pollen grains of fertile and sterile material was performed after obtaining materials with differential male fertility. Compared to being fully and brightly colored in fertile anthers, abnormalities were found in the sterile anthers (Figure 1C(I,IV)). From the SEM (scanning electron microscopy) of pollen grains, it can be revealed that fertile pollen grains are fully rounded and sterile pollen grains are severely collapsed (Figure 1C(II,V)). In the TEM (transmission electron microscopy) observation of mature pollen grains (Figure 1C(III,VI)), a lot of intracellular substances in fertile pollen grains were found, mainly in the form of SG (starch granule), the exine structure of pollen was clearly visible, and the triple layered structure of Te (texine), Ba (bacula) and Ne (nexine) was obvious. In contrast, there was no filling of any content in the sterile pollen grains, Ba intermittently appeared in the exine, and the structural delamination of Ta and Ne was not clear. In addition, the absence of pollen intine was found in male sterile pollen. Therefore, we conclude that the absence of the pollen intine and abnormal pollen exine leads to abnormal starch deposition in the pollen, which contributes to male sterility.

### 2.2. The OsSPL Gene Family Is Involved in Male Fertility Regulation in Response to Temperature Changes

#### 2.2.1. Identification and Phylogenetic Analysis of *OsSPL* Family in Rice

To identify *OsSPL* gene family members, we performed HMMER analysis and a blastp search using the SBP domain and 16 *AtSPL* members, respectively identified as *OsSPL*s in the rice genome [26,27]; as a result, a total of 19 *OsSPLs* were identified. All of the identified OsSPL proteins share the SBP domain. Multiple sequence comparison was performed by the amino acid sequences of the conserved domain of SBP (Figure S1A). To investigate the sequence structure of the *OsSPL*s, we analyzed their motif and intron–exon structure. All the 19 *OsSPLs* share the Motif 3, Motif 2, Motif 1 and Motif 5 structures, forming the conserved SPB functional domain (Figure S2A). This revealed the close evolutionary relationships of the *OsSPL*s. For example, *OsSPL17* and *OsSPL14* are identical in motif structural composition. The exon–intron patterns of the *OsSPL* revealed that *OsSPL9*, *OsSPL15*, *OsSPL1* and *OsSPL6* were in one evolutionary branch and contain the same structure (Figure S2B). The results indicated that the genetic structure of *OsSPL* from closer evolutionary relationships was similar. Then, a phylogenetic tree was constructed based on 19 OsSPLs, 16 AtSPLs, 17 HvSPLs and 10 TaSPLs in the database (Figure S1B). The 62 SPLs were divided into ten groups (I-X). Group IV has the most members, eleven, and the ones occurring least were only two in groups VI. Group II has ten members, groups III and VII have nine members, group I has six members, group IX has five members, and groups V, VIII and X have three members, respectively. Comparison of the SPLs of rice and *Arabidopsis* within the same group revealed that *OsSPL17* and *AtSPL9*, *OsSPL19* and *AtSPL13*, and *OsSPL9* and *AtSPL7* have similar amino acid sequences and physical properties (Table 2). This result suggests that SPLs between rice and *Arabidopsis* may perform similar functions in plant growth and development, and stress tolerance regulation.

#### 2.2.2. Spike Specificity Expression Patterns of *OsSPLs* Showing a Potentiality for Temperature Induced Fertility Regulation in PA64S

**Figure 2:**
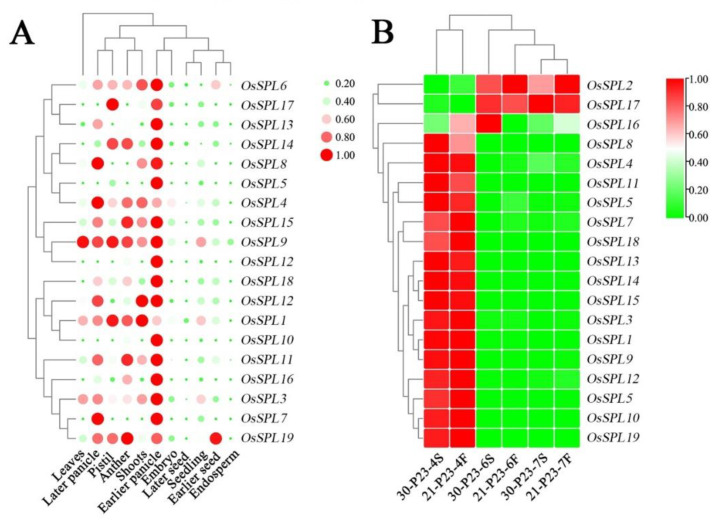
The gene expression analysis of *OsSPL* family in rice tissue. (**A**) Expression profiles of SPL gene family in different periods and tissues of rice. (**B**) Expression profiles of SPL gene family in young spikelets of fertility differential plants treated with different temperatures. 30/21. Treated at 30 °C or 21 °C. P23/P28. PA2364S/PA2864S. 4S/4F. The 4th stage sterile or fertile plants. For each treatment, the RNA-seq results show the average of three replicates.

Based on the Rice Genome Annotation Project database, the tissue specific expression patterns of *OsSPLs* were investigated by the RNA-seq data of nineteen *OsSPLs* in shoots, leaves, seeds, panicle and anthers. As shown, *OsSPL*s were expressed with higher levels in the panicle and anther than in other tissues, and all *OsSPLs* had high expression in the early spikelet (Figure 2A). In addition, some genes (*OsSPL1*, *OsSPL9*, and *OsSPL12*) in seedlings and shoots had high expression. This result indicated that the *OsSPL* gene family is highly expressed in the developmental stages of rice reproductive organs in general and has an important regulatory role in the development of young spikelets and anthers, specifically. We based this conclusion on the standard of the laboratory for many years, and that the critical stage for male fertility transformation is from the third to seventh stages, according to the eight stage differentiation of young spikes. Subsequently, we selected PTGMS rice PA2364S with a fertility transition temperature of 23 °C to obtain fertility differential plants by temperature treatment and performed RNA-seq analysis on spikes at different developmental stages (Figure 2B). The expression levels of *OsSPL* differed significantly among the three young spike differentiation stages of various fertility materials. Among them, *OsSPL2* and *OsSPL17* showed a higher level of expression in the fourth stage, while there were higher expression levels in the sixth and seventh stages. Meanwhile, we found that there were differences in the expression levels of *OsSPL2*, *OsSPL17* and *OsSPL16* after different temperature treatments. This result indicated that fertility transition can be induced by temperature and the expression level of *OsSPL* responds to temperature changes. Therefore, we hypothesize that the spike specific expression pattern of *OsSPL* in rice reveals its potential male fertility regulation ability.

#### 2.2.3. Differential Expressions of *OsSPL* in Sterile and Fertile Panicles of PA2364S and PA2864S under 25 °C Conditions

The fertile and sterile plants of PA2364S and PA2864S in the fifth and sixth stages were subjected to expression analysis. The expression profile illustrated that the *OsSPL* expression levels were higher in the fifth stage panicles from PA2364S and PA2864S at 21 °C, compared with the 30 °C treatment. The panicles *OsSPL* expressions at the sixth stage were consistent with those at the fifth stage (Figure S3). To investigate the specific trends of *OsSPL* genes with temperature treatment, we show their expression in Figure S4. Interestingly, we found that the expression trends of *OsSPL1*, *OsSPL2*, *OsSPL4*, *OsSPL6*, *OsSPL7*, *OsSPL8*, *OsSPL9* and *OsSPL15*, at the two stages, showed lower levels of expression after 30 °C treatment compared with 21 °C treatment. In contrast, the expression trends of *OsSPL10* and *OsSPL16* were opposite, with higher levels under the 30 °C treatment than those at 21 °C. The result is a superimposed expression level of the *OsSPL*s in response to temperature changes and involvement in fertility regulation. It also suggested that the sixth stage was a critical stage for the fertility transition, when the *OsSPL*s might be involved in rice fertility regulation responding to temperature changes.

**Figure 3:**
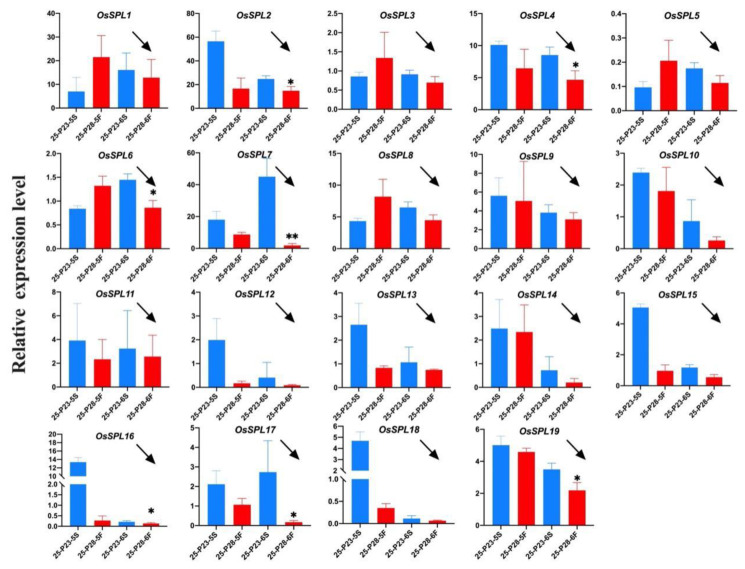
Expressions of *OsSPL* gene family in sterile and fertile panicles of PA2364S and PA2864S under 25 °C treatment. Here, 25: treated at 25 °C. P23/P28: PA2364S/PA2864S. Here, 5S/5F: fifth stage sterile or fertile plants. Arrows indicate the trend of gene expression at the sixth stage. For each treatment, the qPCR results show the average of three replicates. Here, *p* (*) < 0.05, *p* (**) < 0.01.

To verify that *OsSPL* responds to changes in fertility during the critical stage of fertility transition, and exclude the effect of temperature change on *OsSPL* expression, we performed qPCR validation using the two PTGMS rice, PA2364S and PA2864S, selected from the same genetic background, with different fertility transition temperatures. As shown in Figure 3, PA2364S showed male sterility while PA2864S showed male fertility under the same temperature—25 °C treatment. At the sixth stage, all the *OsSPLs* showed increased expression in sterile plants and decreased expression in fertile plants. Among them, the transcription level of *OsSPL2*, *OsSPL4*, *OsSPL6*, *OsSPL16, OsSPL17* and *OsSPL19* differed significantly between the lines, but the greatest differences were observed for *OsSPL7*. This result indicates a regular change in the *OsSPL* expression level with a change in fertility after excluding the effect of temperature changes and the high expression levels in sterile plants. The results further suggest that *OsSPL* genes are involved in the process of fertility regulation in rice.

#### 2.2.4. Differential Expressions of miR156-*OsSPL* Module Genes in Sterile and Fertile Panicles of PA2364S and PA2864S under Different Temperature Conditions

**Figure 4:**
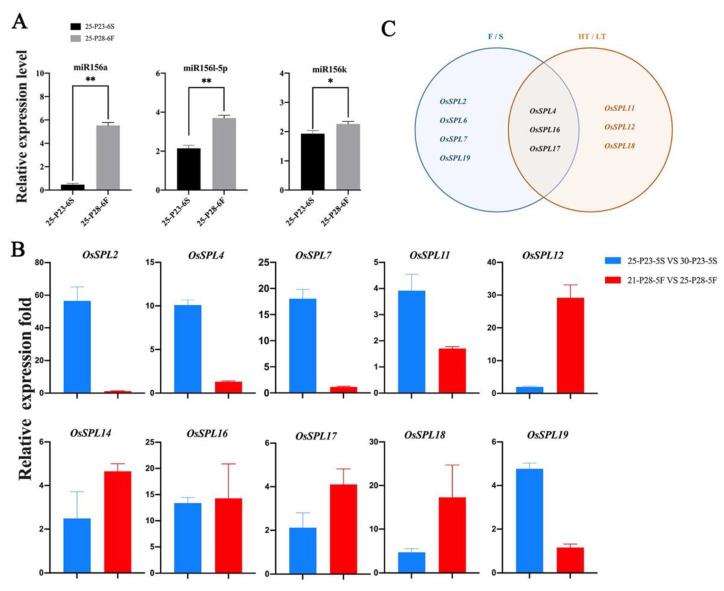
Expression of miR156/*OsSPL* module genes in sterile and fertile panicles of PA2364S and PA2864S at different temperatures. (**A**) Expression levels of miR156a, miR156k and miR156l-5p at sixth stages of 25 °C treatment in PA2364S and PA2864S. (**B**) Relative expression folds of the *OsSPL* genes targeted by miR156 responding to temperature changes. (**C**) Venn diagram of *OsSPL* genes in response to fertility changes and temperature changes. Here, 25: treated at 25 °C. P23/P28: PA2364S/PA2864S. Here, 6S/6F: sixth stage sterile or fertile plants. The blue bars in Figure 4 indicate the fold difference in gene expression between 25 °C and 30 °C treatment in P23 sterile plants, and the red bars indicate the fold difference in gene expression between 21 °C and 25 °C treatment in P28 fertile plants. For each treatment, the qPCR results show the average of three replicates. Here, *p* (*) < 0.05, *p* (**) < 0.01.

Some research has found that the *OsSPL* mediated signaling pathway plays a crucial role in rice meiotic entry though the miR156-*OsSPL* module. According to the relationship network of miRNA and *OsSPLs* identified by our previous studies (Figure S5), a total of nine *OsSPL* genes, including *OsSPL2*, *OsSPL7*, *OsSPL11*, *OsSPL12*, *OsSPL14*, *OsSPL16*, *OsSPL17*, *OsSPL18* and *OsSPL19*, were targeted by miR156a, miR156k and miR156l-5p, respectively. To demonstrate miRNAs-*OsSPLs* module expression, the relative expression levels of miR156a, miR156k and miR156l-5p in PA2364S and PA2864S were also verified by qPCR (Figure 4A). The expression of miR156a, miR156k and miR156l-5p was lower in sterile plants than in fertile ones, after the same temperature—25 °C treatments. The expression trends of these miRNAs were opposite to those of the *OsSPLs*. The result indicated that miR156 negatively regulates *OsSPL* expression and is involved in the regulation of rice fertility. Indirectly, the expression pattern of miR156 demonstrated that SPL plays an important role in the regulation of fertility.

After obtaining the above results, we were interested in the response of miR156 and *OsSPL* to temperature changes. Therefore, we compared the expression patterns of *OsSPL* genes in the fifth stage of plants of PA2364S and PA2864S with temperature changes and unchanged male fertility (Figure 4B). Interestingly, it was found that all the ten *OsSPL* genes (*OsSPL2*, *OsSPL4*, *OsSPL7*, *OsSPL11*, *OsSPL12*, *OsSPL14*, *OsSPL16*, *OsSPL17*, *OsSPL18* and *OsSPL19*) targeted by miR156 showed regular change with temperature. *OsSPL* expression was lower at relatively high temperatures and higher at relatively low temperatures, while the miR156a expression pattern was opposite to them. Among them, *OsSPL4*, *OsSPL11*, *OsSPL12*, *OsSPL16*, *OsSPL17* and *OsSPL18* showed significantly different levels. The results demonstrated that *OsSPL* is sensitive to environmental temperature changes, and miR156/*OsSPL* might be involved in the regulation of male fertility in a modular form, in response to temperature changes. Therefore, following the expression analysis of the gene families, we found that *OsSPL4*, *OsSPL16* and *OsSPL17* might be involved in regulating fertility in response to temperature changes (Figure 4C).

### 2.3. The OsSPL17 Gene Is Highly Homologous with the AtSPL9 Gene and Has the Potential to Regulate Male Fertility

#### 2.3.1. Collinearity and Nonredundant Analysis of the *OsSPL* Family

Gene duplication allows the expansion of new genes with similar or different functions, which is a special mechanism of species evolution. Completing the basic and evolutionary analyses of *OsSPL*s, we analyzed the segmental duplication events in the 19 genes that had been identified. In total, seven pairs of segmental duplication events (*OsSPL2*-*OsSPL16*, *OsSPL2*-*OsSPL18*, *OsSPL4*-*OsSPL11*, *OsSPL5*-*OsSPL10*, *OsSPL3*-*OsSPL12*, *OsSPL14*-*OsSPL17* and *OsSPL16*-*OsSPL18*) were found on the chromosomes 1, 2, 6, 8 and 9, respectively (Figure S6A). It suggested that half of the *OsSPLs* were involved in the segmental duplication events of rice.

To understand the genetic origins and evolution of the *SPL* gene family, genomewide collinearity analysis was also performed. Four representative species were selected with two monocotyledons and two dicotyledons, respectively. The 7 *Arabidopsis* genes, 12 *Hordeum vulgare* genes, 11 *Glycine max* genes and 30 *Zea mays* genes were collinearly related to *OsSPL* (Figure S6B). Meanwhile, we calculated the nonsynonymous substitution/synonymous substitution (Ka/Ks) ratio of the orthologous gene pairs (Figure S6C). The Ka/Ks ratios between rice and *Arabidopsis*, *Hordeum vulgare*, *Glycine max* and *Zea mays* were calculated to be 0.25, 0.40, 0.26, and 0.39. This indicated that *OsSPLs* may have evolved from under strong purifying selection pressure. Analysis of the orthologous relationships between rice and Arabidopsis revealed that seven pairs of their *SPLs* were homologous (Table 3). Among them, *OsSPL2*, *OsSPL16* and *OsSPL19* were homologous to *AtSPL13*, and *OsSPL14* and *OsSPL17* were homologous to *AtSPL9*. By evolutionary analysis, there were seven syntenic *SPL* gene pairs found between rice with *Hordeum vulgare* and *Zea mays*, but no pairs simultaneously among the four species (Figure S6D). The results indicated that the *OsSPL* gene and *AtSPL* gene are homologous genes arising from species differentiation and have similar biological functions.

#### 2.3.2. *OsSPL17* Has a Similar Protein Structure to AtSPL9 and Is a Core Gene in the Male Fertility Related Module

**Figure 5:**
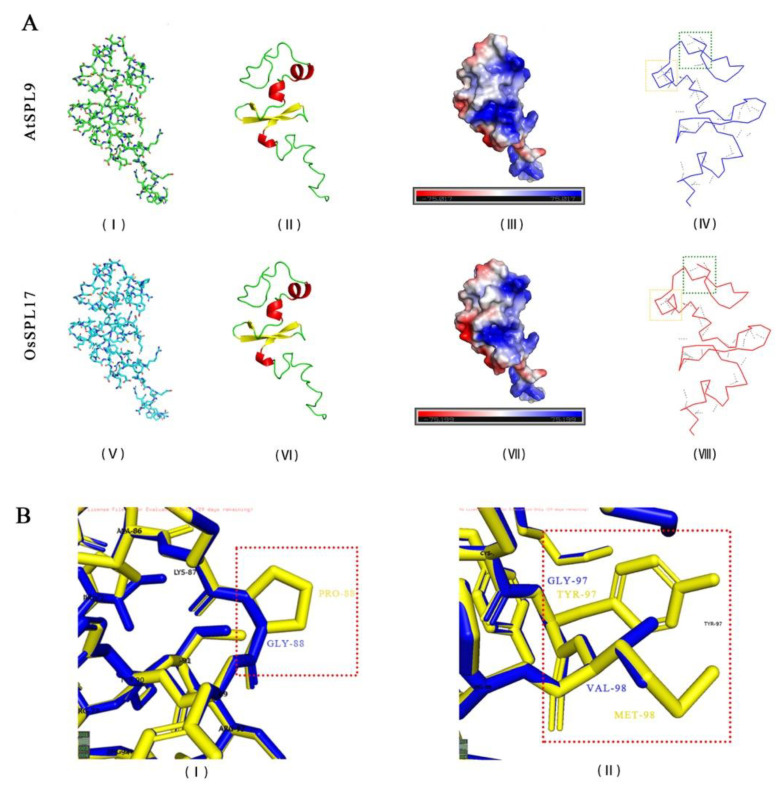
Comparative analysis of AtSPL9 and OsSPL17 protein structures. (**A**) The stick structure (**A**(**I**,**V**)), secondary structure (**A**(**II**,**VI**)), surface electrostatic potential (**A**(**III**,**VII**)) and hydrogen bonding structure (**A**(**IV**,**VIII**)) of AtSPL9 and OsSPL17 proteins. (**B**) Differences in amino acid composition and protein structure between OsSPL17 and AtSPL9. In (**A**(**II**,**VI**)), the red part indicates the α-helix and the yellow part indicates the β-fold. In (**A**(**IV**,**VIII**)), the gray dashed lines indicate hydrogen bonds. In (**B**), the yellow and blue structures indicate the amino acid arrangement of AtSPL9 and OsSPL17, respectively. PRO: Proline. GLY: Glycine. TYR: Tyrosine. MET: Methionine. VAL: Valine.

OsSPL4, OsSPL16 and OsSPL17 were found to have important roles in regulating male fertility in response to temperature changes, according to the results of expression analysis. Integrating the homology between rice and Arabidopsis, we explored the protein structures of OsSPL17 and AtSPL9 to determine whether they have similar biological functions. Previous studies have found that *AtSPL9* can regulate anthocyanin biosynthesis genes and control anthocyanin and flavonoids content in plants, and the flavonoids are necessary for the formation of male fertility in rice [28,29]. The comparison revealed that AtSPL9 and OsSPL17 have similar protein backbones, which have a variation of amino acids only in the chain (Figure 5A). They have identical α-helix and β-fold structures in the secondary structure. Hydrogen bonding is the force that maintains the secondary structure of the protein, and more hydrogen bonding structures were found in AtSPL9 by comparison (Figure 5A(IV,VIII)). Local structural comparisons revealed that the increase in hydrogen bonding was due to differences in the amino acids at some positions. For instance, PRO at position 88 in AtSPL9 and GLY at position 88 in OsSPL17 (Figure 5B(I)). Similarly, there are amino acid differences in TYR/GLY and MET/VAL at positions 97 and 98 between AtSPL9 and OsSPL17 (Figure 5B(II)). In addition, they have similar protein surface structures and surface electrostatic potentials. The results showed that OsSPL17 and AtSPL9 have similar compositions and structures of proteins, and they have a greater degree of similarity in biological functions.

**Figure 6:**
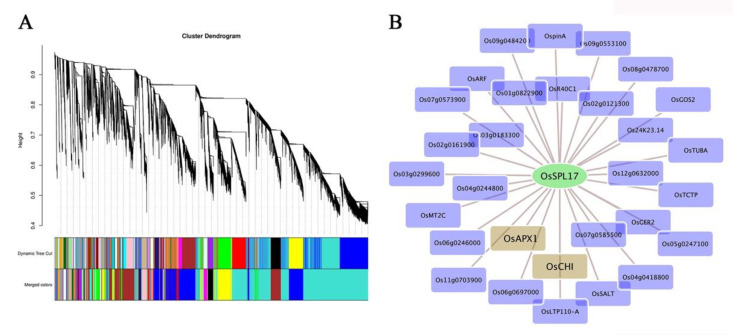
Weighted gene co-expression network in PA64S. (**A**) Hierarchical cluster tree. (**B**) Gene co-expression network of the darkturquoise module. Green indicates the core genes in the module. Brown color indicates the genes that interact with the core genes in our module of interest.

To investigate the potential role of *OsSPL17* in the regulation of male fertility in rice, we performed WGCNA of the gene expression data from the obtained transcriptome. We clustered the genes into 30 related modules via association with male fertility traits (Figure 6A). After further analysis, we correlated the fertility related module with *OsSPL17*, which was finally identified in the darkturquoise module (Figure 6B). Several core genes were identified in the darkturquoise module, including *OsSPL17*. In particular, *CHI* and *APX1* were identified among the co-expression genes of *OsSPL17*, which are critical genes for flavanone synthesis in the flavonoid pathway and major antioxidant enzymes in the ROS (reactive oxygen species) scavenging pathway, respectively. Flavonoids are inevitable for the formation of male fertility in rice. Meanwhile, our previous research found that, following the abnormal degradation of the tapetum in sterile anthers, ROS are essential for the initiation of the PCD process in the tapetum [30]. Therefore, the results indicated that *OsSPL17* has the potential to regulate male fertility.

### 2.4. OsSPL17 Is Involved in Flavonoid Metabolic Processes and Regulates Male Fertility in PA64S

**Figure 7:**
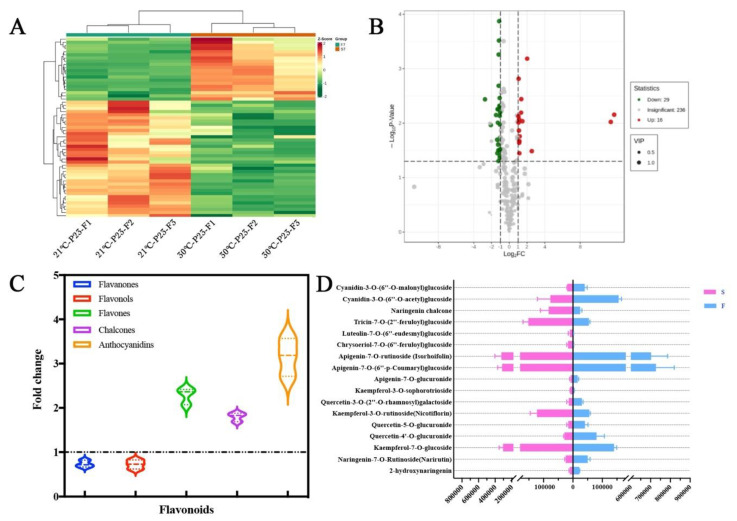
Analysis of flavonoid metabolites in anthers of fertility differential plants. (**A**) Cluster heatmap of differential metabolites. (**B**) Volcano Plots of differential metabolites. (**C**) Ratio of total class II flavonoid metabolites in fertile to sterile anthers. (**D**) Relative contents of different class II flavonoid metabolites in fertile and sterile anthers.

The miR156/*SPL9* module can negatively regulate flavonoid biosynthesis in Arabidopsis [29]. OsSPL17 was found to have a similar protein structure to ATSPL9 and a gene interaction relationship in regulating the flavonoid pathway from evolutionary analysis, protein structure analysis and co-expression analysis. To investigate the regulatory role of *OsSPL17* in flavonoid metabolism, we performed the metabolomic analysis of flavonoid substances in the fertility differential material. Significant differences in flavonoid metabolites between fertile and sterile plants were found in the analysis of flavonoid metabolites of fertile differential plants. Among the significantly different metabolites, 16 flavonoids were up regulated and 29 were down regulated in sterile plants, respectively, compared to fertile plants (Figure 7B). The content of several major class II flavonoids was different in the fertility differential material, such as flavanones, flavonols, flavones, chalcones and anthocyanidins. The ratio of the content in the fertile compared with the sterile plants showed that the flavanones and flavonols were higher in the fertile plants and the opposite results were obtained in the flavones, chalcones and anthocyanidins (Figure 7C). From the differentially expressed class II metabolites, we found that two flavanones, 2-hydroxynaringenin and narirutin, were up regulated in the expression of fertile plants, and kaempferol-7-O-glucoside and nicotiflorin are expressed as down regulated in fertile plants. Among the six flavones substances, apigenin-7-O-glucuronide, apigenin-7-O-(6′′-p-Coumaryl) glucoside and Isorhoifolin were up regulated in fertile plants, while the other three (chrysoeriol-7-O-(6′′-feruloyl) glucoside, luteolin-7-O-(6′′-eudesmyl) glucoside and tricin-7-O-(2′′-feruloyl) glucoside) were down regulated in fertile plants (Figure 7D). The results showed that the substance composition of the different class II flavonoid metabolites was quite different in the fertile differential material, further suggesting that flavonoid metabolic processes may be regulators of male fertility in rice.

**Figure 8:**
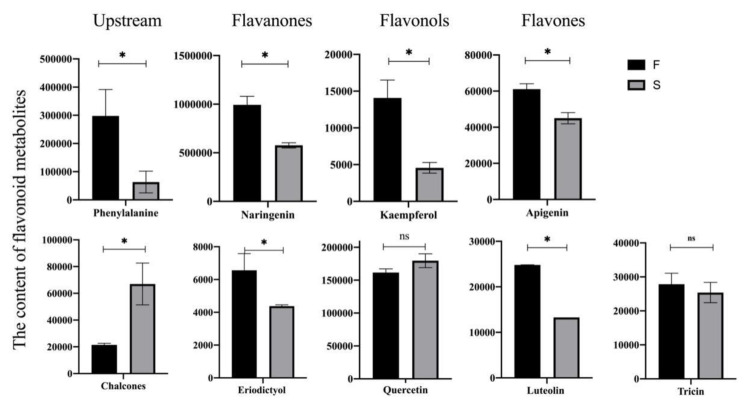
Relative flavonoid content of class II flavonoids in fertility differential plants. Sterisks indicate significant differences revealed by Student’s *t*-test at *p* < 0.05 (*) and ns. Not significant.

To further investigate the function of flavonoids in rice fertility, we extracted the major fractions of the different class II flavonoid metabolites in the anthers of fertile differential plants. Naringenin and eriodictyol substances were mainly extracted from flavanones, and they were both present in higher levels in fertile plants compared to sterile plants (Figure 8). Meanwhile, we also detected flavonols (kaempferol and quercetin), flavones (apigenin, luteolin and tricin) and chalcones. Apart from quercetin and chalcones, all substances were up regulated in fertile plants. This result indicates that fertile rice anthers have high levels of flavonoids, which are essential for the formation of male fertility in rice.

**Figure 9:**
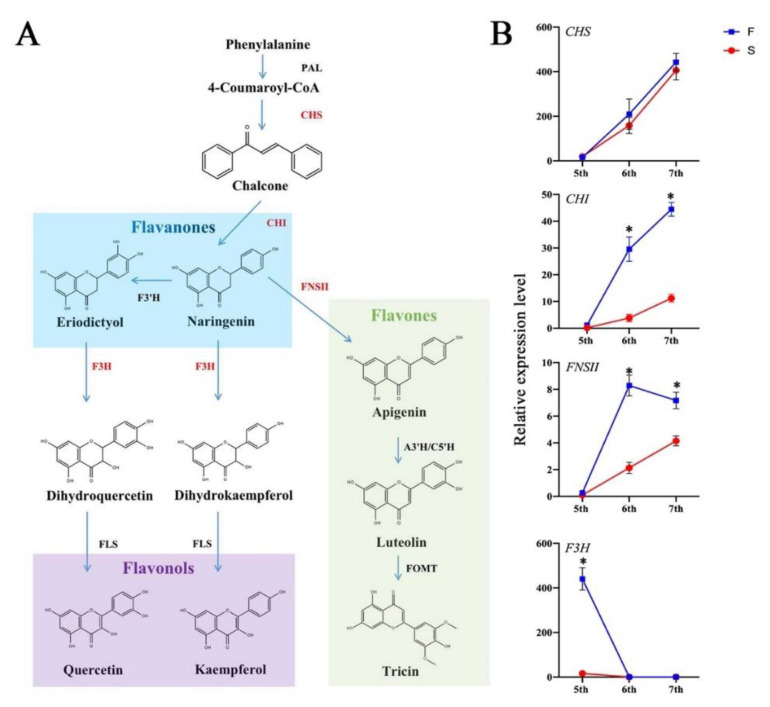
Rice flavonoid biosynthesis pathways and expression of genes encoding key enzymes. (**A**) Rice flavonoid biosynthesis pathway. (**B**) The relative expression level of genes encoding key enzymes in PA2364S. CHS, chalcone synthase; CHI, chalcone isomerase; F_3_H, flavanone 3-hydroxylase; FLS, flavonol synthase; FNSII, flavone synthase II; A3′H/C5′H, apigenin 3′-hydroxylase/chrysoeriol 5′-hydroxylase; FOMT, flavonoid O-methyltransferase. Sterisks indicate significant differences revealed by Student’s *t*-test at *p* < 0.05 (*).

To investigate the process of flavonoid biosynthesis in rice anthers, we presented a flavonoid metabolic pathway and performed expression analysis of several key enzyme genes in the biosynthesis of important class II flavonoid (Figure 9). *CHS* is the first branch point enzyme encoding the biosynthesis of flavanones and the expression in fertile plants was slightly higher than that of sterile plants. The expression levels of *CHI* and *FNS2* were higher in fertile plants than that in sterile plants at three stages (fifth, sixth and seventh), and *CHI* showed significantly up regulated expression in the sixth and seventh stages. This result demonstrated that there was a vigorous flavonoid biosynthesis process in the rice anthers of fertile plants. Interestingly, a higher expression of *CHI* and *FNS2* genes was observed in the later stages of young spike development; these results indicate that flavonoid substances start efficiently synthesized at the sixth stage. *F3H* is a gene encoding the synthase of flavonols and its expression is higher in the early stages of young spike development. However, the contents of kaempferol and qurerrtin were inconsistently expressed in fertile plants. Therefore, the results speculated that the flavonoids involved in fertility regulation are mainly flavonols and flavones. *OsSPL17* negatively regulate *CHI* to increase the biosynthetic process of flavonols and flavones, which provide more flavonoid substances for fertile plant fertility formation.

### 2.5. Regulated by OsSPL17, Delayed ROS Accumulation Causes Delayed PCD of the Tapetum in PA64S

**Figure 10:**
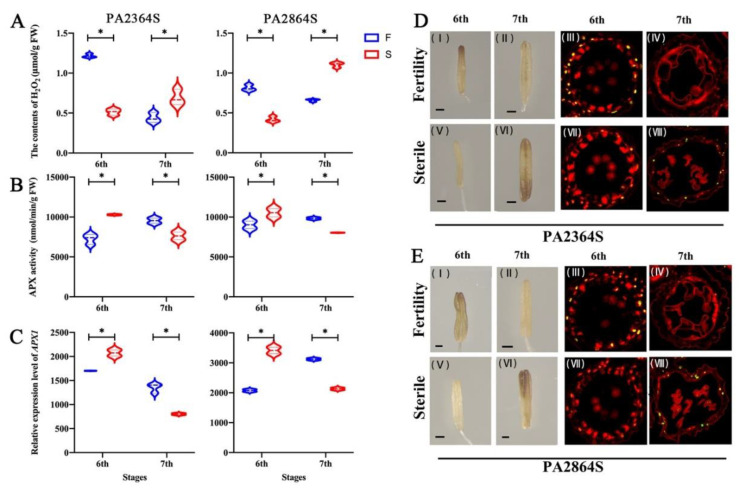
Effect of ROS and APX on the PCD process in the anther tapetum. (**A**) The content of H_2_O_2_ in PA2364S and PA2864S. (**B**) The APX activity in PA2364S and PA2864S. (**C**) The relative expression level of *APX1* in PA2364S and PA2864S. (**D**) DAB staining and transferase mediated dUTP nick end labeling (TUNEL) assay of PA2364S (**D**) and PA2864S (**E**) anthers. Sterisks indicate significant differences revealed by Student’s *t*-test at *p* < 0.05 (*).

ROS homeostasis is critical for cell survival and growth during anther development, especially for timely PCD in the tapetum [31,32]. APX (Ascorbate peroxidase) is a major scavenger of ROS, which converts H_2_O_2_ to H_2_O and balances the ROS level. To investigate how the *OsSPL17* regulates *OsAPX1* expression and regulates the male sterility process, we measured the H_2_O_2_ content and APX enzyme activity in rice anthers. The results showed that H_2_O_2_ was higher in fertile plants at the sixth stage and higher in sterile plants at the seventh stage among PA2364S and PA2864S (Figure 10A). It was found that the anthers stained darker at stage six in fertile plants and at stage seven in sterile plants with DAB staining, and this result is consistent with the measured value of H_2_O_2_ content (Figure 10C). To further investigate whether the ROS content in the tapetum is consistent with the anther phenotype, we performed a TUNEL assay on anther sections. There was a clear fluorescence signal in the tapetum region in the sixth stage of the PA2364S fertile anthers (Figure 10C(III)), compared with a weaker fluorescence signal in the tapetum of sterile anthers (Figure 10C(VII)), which suggested that there were strong ROS accumulation and PCD processes in the tapetum of fertile anthers. In contrast, the opposite phenomenon was observed in the seventh stage, and a stronger fluorescence signal appeared in the tapetum of sterile anthers (Figure 10C(IV,VIII)). This variation pattern was also demonstrated in PA2864S (Figure 10D). The results indicated that the ROS accumulated earlier and induced the PCD process in the tapetum of fertile anthers during anther development. The ROS accumulated later in the tapetum of sterile anthers and delayed the PCD process. To determine the relationship between ROS changes and APX, we measured APX activity in anthers (Figure 10B). The results showed that APX activity was higher in fertile plants at stage six than in sterile plants, and the opposite result was obtained at stage seven. Meanwhile, the expression pattern of *APX1* gene in PA2364S and PA2864S was consistent with the trend of enzyme activity (Figure 10C). Therefore, we concluded that *OsSPL17* interacts with *APX1* to affect APX activity in the anther, thereby regulating ROS content in anthers and the tapetum, then controlling the PCD process in the tapetum at last regulating male fertility in rice.

## 3. Discussion

### 3.1. Function of SPL Family Genes in Rice

SPL transcription factors are a family of proteins with a highly conserved SBP structural domain unique to photosynthetic plants [5]. Up to now, the *SPL* gene family members have been identified in various plants, including *Arabidopsis*, *Oryza sativa*, *Triticum aestivum* and strawberry (*Fragaria vesca*) [2,15,33,34,35,36,37]. The number of *SPL* gene family members varies widely among species [38,39]. In this research, there were 27 alternative splicing *OsSPLs* sequences identified during the initial process, mainly in the *OsSPL1*, *OsSPL4*, *OsSPL12* and *OsSPL16* sequences. We selected the maximum length alternative splicing sequences for subsequent gene family identification and sequence alignment, and a total of 19 *OsSPLs* were identified in rice. Multiple segmental duplications, tandem duplication and transposition events are major drivers of genome and genetic system evolution [40,41]. In the evolutionary relationships of *SPL* genes, five homologous gene pairs of Oryza sativa were identified to locate within segmental duplication region, and this result was also reported in the research of Zhong et al. [42]. Meanwhile, the number of collinear genes was lower in rice with *Arabidopsis* and *Glycine max* (7 and 12). Notably, we found seven shared *OsSPL* genes with collinearity in three species other than *Arabidopsis*, and the results suggest that the orthologous correlations between these species are relatively conserved during evolution, and these *OsSPL* genes might present before species divergence [43]. The Ka/Ks of collinear gene pairs were lower than 1 between rice and the four species, and the results suggest that there might have been a stronger purifying selection pressure of the *SPL* gene family in species evolution. Previous studies indicated that the *SPL* gene family is widely involved in plant growth and development and stress responses [44,45]. *AtSPL3*, *AtSPL4* and *AtSPL5* could promote flowering in Arabidopsis by directly activating the expression of *AP1*, *LFY*, and *FUL* [46]. The reports in the literature indicate that *SPL* is involved in the reproductive growth process and is essential for fertility determination [47,48]. The loss of function spl8 mutant showed reduced fertility, which was mainly characterized by abnormal pollen sacs and reduced pollen production [49]. In addition, *SPL* could act as a regulatory center in response to environmental temperature. The miR156-*SPL3* module could respond to environmental temperature changes by controlling *FT* expression to regulate the flowering process. In addition, the miR156-*SPL9*-*SOC*1 regulatory module also has the ability to control plant flowering in response to temperature changes [50,51]. In the homologous gene relationship analysis, *OsSPL17* and *OsSPL14* were orthologous to *AtSPL9*, and they constituted selected pressure for purifying selection. Similarly, *OsSPL2*, *OsSPL16* and *OsSPL19* were orthologous to *AtSPL13*. However, we observed that *AtSPL9* has a key role in regulating flavonoid metabolism and plant immune response, and we hypothesized that *OsSPL17* and *OsSPL14* have similar functional roles with *AtSPL9* in plant development, focused on the gene function of *OsSPL17* in the study.

### 3.2. OsSPL Responds to Temperature Changes and Is Involved in the Regulation of Male Fertility in PTGMS Rice

In general, the function of genes depends on their expression levels in plants [52]. The expression level of *SPL* genes are regulated by miRNA targeting, in general, and plays a key role in plant embryo, tissue, hormone response and stress response [53]. In this study, we selected two rice varieties (PA2364S and PA2864S) with the same genetic background and different fertility transition temperatures bred by our research team [54], and the expression levels of the identified *OsSPL* genes in PA2364S and PA2864S were analyzed. It was found that the expression level is a superimposed expression of the gene in response to fertility and temperature treatment. PA2364S was male sterile and PA2864S was male fertile under 25 °C treatment. Therefore, we could obtain the *OsSPL* gene expression levels of differentially fertile plants under 25 °C treatment. At the critical stage of fertility transition (the sixth stage), the expression levels of *OsSPL* in differentially fertile plants were significantly different (*OsSPL2*, *OsSPL4*, *OsSPL6*, *OsSPL7*, *OsSPL16*, *OsSPL17* and *OsSPL19*), and the expression levels of *OsSPL* in sterile plants were higher than those in fertile plants. The results indicated that the *OsSPL* gene is involved in regulating male fertility in rice. A significant reduction in setting rate was observed in *OsSPL2*, *OsSPL7*, *OsSPL16* and *OsSPL17* by knocking out 19 SPL family genes in rice [55]. Ren et al. revealed that *OsSPL* may act upstream of *MIL1* (*MICROSPORELESS1*) and *MIL2* (*MICROSPORELESS2*) to regulate meiotic entry and parietal cell differentiation in rice [25]. It is well known that genes are regulated by miRNA targeting, and the expression of most *BpSPL* in terminal buds and male inflorescences was also observed to be negatively correlated with miR156 expression in Betula (*Betula platyphylla* Suk) [56]. We found that miR156 (miR156a, miR156k, miR156l-5p, miR156j-3p and miR156f-3p) targets 10 *OsSPL* genes in our material, after analyzing the target relationships of miRNAs by sequencing (Figure S5). The expression analysis of miR156 in plants treated at 25 °C revealed a low relative expression in sterile plants, and this result contrasted with the expression trend of *OsSPL*. Fifty-seven percent of miR156 targeted *SPL* in *Zea mays* was inhibited by salt and drought stresses, and the results suggest that miR165/*SPL* modules might be jointly involved in regulating growth and development in response to environmental changes [36,57]. Regulatory modules consisting of miRNAs and target genes have been reported in response to disease resistance and abiotic stresses in plants. Some common targeting *OsSPL* genes exist in miR156/529 that have important roles in regulating inflorescence structure and seed yield in rice [58]. For instance, panicle architecture and grain size were controlled by miR529a through altering the expression of all five target genes *OsSPL2*, *OsSPL7*, *OsSPL14*, *OsSPL16*, *OsSPL17* and *OsSPL18* [59]. Overexpression of miR156k resulted in the down regulation of *SPL3*, *SPL14* and *SPL17*, which reduced tolerance to cold stress in rice [60]. To explore whether *OsSPL* genes have a unique pattern in response to temperature changes, we compared *OsSPL* expression levels in the fifth stage plants of two varieties treated with different temperatures. Interestingly, all 10 *OsSPL* genes targeted by miR156 showed a lower expression level in the relatively high temperature treatment (30/25 °C) and a higher expression level in the relatively low temperature treatment (25/21 °C), while the opposite trend of miR156 expression was observed in fifth stage plants, among them, *OsSPL4*, *OsSPL11*, *OsSPL12*, *OsSPL16, OsSPL17* and *OsSPL18* were significantly different levels. The results indicate that miR156 is an miRNA that responds to environmental temperature changes, which is consistent with the previous research that miR156 is upregulated in expression by high temperature conditions [61]. Temperature changes were not high temperature stress or low temperature stress in this study, and the results illustrated that the miR156/*SPL* module is more sensitive in response to temperature changes. *OsSPL4*, *OsSPL16* and *OsSPL17* were revealed to be significantly differentially expressed in response to both temperature and fertility changes; they might be more important in responding to temperature and regulating male fertility. We speculated that the difference in fertility critical temperatures, between PA2364S and PA2864S, might be a change in the expression level of *OsSPLs* in response to temperature, and, thus, the change in male fertility in rice.

### 3.3. OsSPL17 Is Involved in Male Fertility Regulation by Impacting Flavonoid Metabolic and Tapetum PCD Processes

**Figure 11:**
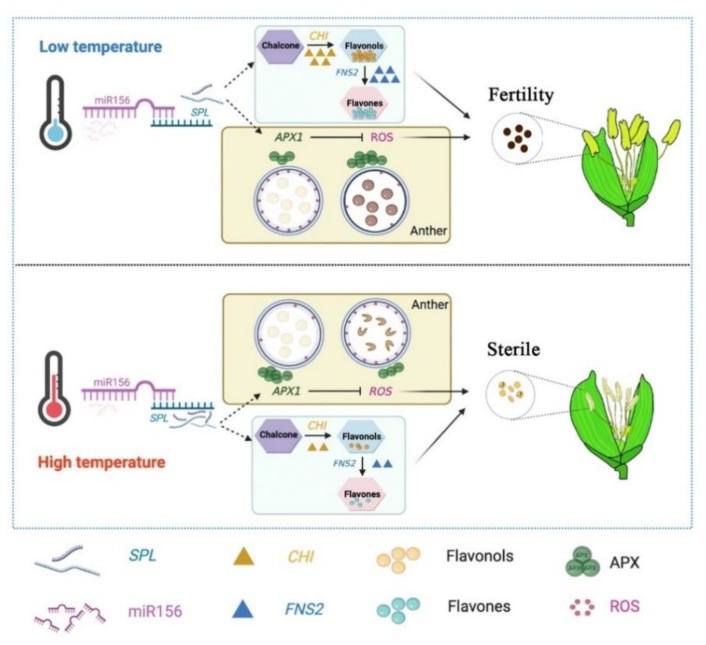
A model for *OsSPL* response to temperature change and involvement in the regulation of rice fertility. Under low temperature conditions, the miR156-*OsSPL* module regulated *CHI* expression in response to temperature changes, resulting in a strong synthesis of flavonols and flavones in the flavonoid pathway, providing sufficient flavonoid substances for the formation of fertile pollen. In addition, the miR156-*OsSPL* module regulates *APX1* gene expression and controls the PCD process in the tapetum by affecting the APX-ROS balance, providing the material basis for the formation of fertile pollen.

Flavonoids are a common class of plant secondary metabolites with a variety of regulatory roles in plant physiological responses, growth and development [62]. They are mainly classified into six class II flavonoids: flavanones, flavonols, flavones, chalcones, anthocyanidins and proanthocyanidins, and more than 6000 flavonoids have been identified [63]. Studies have demonstrated that flavonoids play a regulatory role in biotic and abiotic stresses; in addition, the functional role of flavonoids in male fertility has been demonstrated in several plants [64,65,66,67]. Flavonoids are essential for the formation of male fertility in rice [68]. The miR156-SPL9 module was reported to regulate the flavonoid biosynthetic pathway in Arabidopsis, and *SPL9* upregulation affected flavonol biosynthesis and inhibited flavonol accumulation [29]. In our study, we found that rice *OsSPL17*, which responds to temperature changes and regulates the fertility process, and AtSPL9 in Arabidopsis are proteins of the same subfamily, with some structural and functional similarities. In addition, the *CHI* genes interacting with *OsSPL17* were found to be involved in the regulation of the flavonoid synthesis pathway in WGCNA analysis. The analysis of flavonoid metabolites revealed that the composition of the different class II flavonoids differed significantly in the fertile differential material. Flavonols showed a significant up-regulation of the main substance, kaempferol, in fertile plants, although the total amount was low in comparison with sterile plants. In addition, we found that flavanones and flavones were also significantly upregulated in fertile plants, which indicated that flavonoid substances were at higher levels in fertile plants. Interestingly, we detected that the upstream metabolites of flavonoids, chalcone was significantly down-regulated in fertile plants, and the change in phenylalanine was the opposite. Phenylalanine is an important substance in the phenylpropanoid metabolic pathway, and its content changes directly affect the metabolic processes of downstream flavonoids and lignin. However, the process of lignin metabolism is indispensable for sporopollenin synthesis during male fertility development [28]. Analysis of the expression of genes encoding key enzymes in the flavanones, flavonols and flavones biosynthetic pathway revealed that the expression of *CHI* and *FNS2* were at a high level in fertile plants, especially at the early stage of anther development. Active flavonoid biosynthesis processes are present in fertile plants, which were directly responsible for the high content of flavanones and flavones. Some studies found that *MYB* (*MYB11*, *MYB12* and *MYB111*) regulates the expression of several early flavonoid biosynthetic genes, including *CHS*, *CHI*, *F3H* and *FLS1*, while *SPL* may have an indirect effect on *MYB12* and *MYB111* via a feedback loop [69,70,71]. Therefore, it was hypothesized that *OsSPL17* might affect rice male fertility by regulating the accumulation process of flavonoid substances (Figure 11).

The development and maturation of male gametes play an important role in the fertility and reproduction of rice. Pollen development is a multisystem coordinated and complicated physiological process [72]. The anther is an important site for pollen development, which is composed of four layers of sporophytic cells (epidermis, endothecium, middle layer and tapetum) from outside to inside [73]. The tapetum is the innermost structure of the anther and contributes to microspore release, nutrient accumulation, pollen wall synthesis, and sporopollenin deposition [74,75]. Controlled degradation of the tapetum is essential, and changes in this process can directly reduce fertility [76]. It has been found that the delayed degradation of the tapetum causes male sterility in rice [72,77,78]. In this study, we found that the PCD process normally starts at an early stage in the tapetum cells of fertile plants, whereas that process in the tapetum of sterile plants starts at a later stage of development (Figure 10D,E). Similarly, the accumulation pattern of ROS in anthers was also consistent with its variation pattern (Figure 10A). ROS are involved in the PCD process in the tapetum, and timely PCD in the tapetum and the maintenance of dynamically balanced ROS levels are essential for anther cell growth and survival. The co-expression between *APX1* and *OsSPL17* was found in the WGCNA analysis, and the expression of *APX1* and APX enzyme activity assay revealed that the expression pattern was opposite to the ROS accumulation pattern: low ROS accumulation was observed during the stage of high APX activity in anther the tapetum (Figure 10B,C). In addition, Liu et al. found that the down regulation of APX and GPX activity increased the accumulation of ROS in late pollen development in wheat male sterile materials [79]. In addition, TEM observation of mature pollen grains revealed the absence of starch grain formation, the absence of pollen intine structure and abnormal pollen exine structures in sterile pollen grains (Figure 1C). The tapetum has a secretory role during sporogenesis. Accompanying callus cell degradation, a large amount of sporopollenin precursor material derived from the tapetum is stored on the primexine, and constitutes the basic structure of the pollen exine. *GAMYB*, *CYP703A3* and *GPT1* were demonstrated to be essential for pollen exine development in rice, and these mutants all showed abnormal PCD in the tapetum. Therefore, we propose that *OsSPL17* may impact APX enzyme activity by regulating the expression of *APX1*, which regulates the balance of ROS in the anther tapetum, leading to abnormal PCD processes in the tapetum and producing male sterility (Figure 11).

## 4. Materials and Methods

### 4.1. Plant Materials and the Temperature Experimental Treatment for Pollen Fertility Transformation

The near isogenic lines (NILs) PA2364S and PA2864S come from Peiai64S (PA64S) used in the study are PTGMS rice, which had been identified and bred by our research team for multiple generations. PA2364S has a fertility critical temperature of 23 °C, and, under long day (LD) conditions, male sterility manifested when the average temperature was above 23 °C, and males were fertile at average temperatures below 23 °C. PA2864S has a fertility critical temperature of 28 °C, and, under LD conditions, male sterility manifested when the average temperature was above 28 °C, and males were fertile at average temperatures below 28 °C. This experiment was conducted at the Crop Physiology and Production Centre of Huazhong Agricultural University (30.28° N, 114.20° E) during 2020–2021. Sowed annually on May 10, better growing seedlings were selected and transplanted into enamel pots to grow naturally. Three plants were transplanted into each pot, each limited to primary tillers. During this period, regular fertilizer, water and disease management was performed. According to the method of eight stage differentiation of young rice spikes [80], when the plant population (50%) develops into the secondary stalk and spikelet primordium differentiation stage (3rd stage), the rice plants were moved to the plant growth chamber. The average temperatures were 21 °C, 25 °C and 30 °C with fluorescent light of 300 μmol/(m^2^·s) and relative humidity of 80% of PA2364S and PA2864S.

### 4.2. Phenotype and Characterization Analysis of PTGMS Rice

The representative mature rice plants and spikelets were selected for observation according to our previous research methods [13,81]. The anthers of the top floret that had initiated heading were picked and stained with 1% I_2_-KI. The anthers were extruded with tweezers to remove the residue, and three fields of view were observed randomly under the microscope. Pollen was classified into two categories, fertile and abortive (pollenless, round abortive and spot abortive), according to their different morphologies, and the percentage of fertility was calculated. Finally, the percent fertility of all tested spikelets from the same treatment was averaged to obtain the percentage of pollen fertility for the period under the treatment. We selected 60 plants with more than two young spikelets per plant for observation, with pollen fertility and seed setting rate (N > 120). Significant differences are, according to Student’s *t*-test, at *p*  <  0.05 (*).

### 4.3. Identification and Bioinformatics Analysis of SPL Genes in Rice

To identify members of the *SPL* gene family in rice, the rice genome and genome annotation files were downloaded from Ensembl Plants release 50 (http://plants.ensembl.org/index.html, accessed on 5 July 2021) and that of SBP domain (PF03110) from the Pfam database [27]. First, we used hmmsearch (http://www.hmmer.org/, accessed on 5 July 2021) with SBP domain to search the poplar amino acid sequences, with a threshold of e < 1 × 10^−5^. We obtained all the *Arabidopsis* SBP-box gene family (AtSPL) protein sequences from the TAIR database (https://www.arabidopsis.org/index.jsp, accessed on 11 July 2021) by querying the sequences. Based on these data, the sequences of the most representative *SPL* gene family members in rice were extracted using the TBtools [82], and the OsSPL protein sequence was further queried and validated by BLASTp in the NCBI protein database. (https://blast.ncbi.nlm.nih.gov/Blast.cgi?PROGRAM=blastp&PAGE_TYPE=BlastSearch&LINK_LOC=blasthome, accessed on 13 July 2021). We used Batch CD-Search in NCBI for further screening based on conserved structural domains to obtain candidate genes, which were also removed (https://www.ncbi.nlm.nih.gov/Structure/bwrpsb/bwrpsb.cgi, accessed on 15 July 2021). The ExPASy website (http://expasy.org/tools/, accessed on 15 July 2021) was employed for evaluations of molecular weight (MW), isoelectric point (pI) and amino acid numbers of the identified OsSPL proteins [83].

The physical location of the *OsSPL* gene was obtained from the genome annotation information of rice, the visualization of the *OsSPL* gene on the rice chromosome was performed using TBtools, and the intron–exon structure was demonstrated. Conserved motifs of the OsSPL protein were identified using MEME v5.4.3 (https://meme-suite.org/meme/tools/meme, accessed on 22 July 2021) with the maximum motif number set to 20. Finally, the conserved amino acid sequences were visualized using the WebLogo online tools (http://weblogo.berkeley.edu/, accessed on 22 July 2021).

The SPL protein sequences of rice, *Arabidopsis*, *Hordeum vulgare* and *Triticum aestivum* were used to construct phylogenetic analyses [84,85], and multiple sequence comparisons were performed and an unrooted phylogenetic relationship was constructed by MEGA 7.0 software, the phylogenetic relationship was constructed by the Jones–Taylor–Thornton (JTT)+ gamma distributed (G) model based on 1000 bootstrap replicates [86]. Finally, the images were processed using Figtree v1.4.4 software and Adobe illustrator 2021.

Genomic and annotation files of *Arabidopsis*, *Zea mays*, *Hordeum vulgare* and *Triticum aestivum* were obtained from the Ensembl database for species collinearity analysis. Using the TBtools with MCScanX, we analyzed the tandem duplication events and the collinearity relationship for gene pairs from different species [87]. Ka and Ks substitutions between gene pairs were also calculated, by use of the TBtools [82].

### 4.4. RNA Sequencing and qPCR Validation for PA2364S and PA2364S Rice

The 4th, 6th and 7th stage materials of PA2364S and PA2864S were selected for the extraction of total RNA, using TIANGEN RNAprep Pure Plant Kit as described by the supplier. For RNA sequencing, 3.0 μg of RNA from each sample was used for sequencing. Each sample was represented by three biological replicates.

For mRNA quantification, the 5th, 6th and 7th stages materials of PA2364S and PA2864S were selected for the extraction of total RNA, and followed the instructions of the RevertAidTM First Strand cDNA Synthesis Kit (MBI, Lithuania) to reverse transcribe RNA samples into cDNA. In this experiment, Acting7 (X16280) was selected as the internal reference gene. We used primer 3.0 to design specific primers, and these primers were synthesized by Shanghai Shenggong Bioengineering Co., Ltd., Shanghai, China. The specific primer information is shown in Supplementary Table S1. The QuantStudio™ Real-Time PCR Detection System was used for qPCR, and each sample was represented by three biological replicates. For miRNA quantification, according to the instructions, we used a TIANGEN miRcute enhanced miRNA cDNA first-strand synthesis kit to obtain reverse transcription products in a PCR machine (Bio-Rad, Hercules, CA, USA). A TIANGEN miRcute Enhanced miRNA Fluorescence Quantitative Detection Kit (SYBR Green) was used for qPCR of miRNA. U6 was selected as the internal reference gene. The expression of miRNA was verified by three biological replicates. Values were represented as the average values of three biological repeats. Error bars represented standard deviations. Asterisks indicated significant differences revealed by the Student’s *t*-test.

### 4.5. Analysis of the Protein Structure and WGCNA of PA64S

Analysis of protein structures was performed using SWISS-MODEL and Phyre2 to ensure their credibility [88,89]. Files constructed in the database were visualized and edited using PyMOL (The PyMOL Molecular Graphics System, Schrödinger, LLC., New York, NY, USA) software.

The co-expression network was constructed using the WGCNA package in Rsoftware. In this study, the data from 18 samples were analyzed. Before performing WGCNA analysis, selected genomes were filtered to remove low quality genes, and the data were filtered by setting a coefficient of variation of 0.5. The parameters of WGCNA program were as follows: power estimate (estimate value) = 8, min module size = 30, merge cut height = 0.25. The other parameters were defined as default values. Highly similar modules were subsequently identified by clustering and then merged into new modules on the basis of eigengenes. Finally, Cytoscape 3.8.2 was used to visualize the data.

### 4.6. Electron Microscopy Methods and TUNEL Assays of PA64S

The sample preparation and observation for TEM (transmission electron microscopy), SEM (scanning electron microscopy), and TUNEL (transferase mediated dUTP nick end labeling) of rice anthers were performed according to the previous work [77].

### 4.7. Determination of DAB Staining, H_2_O_2_ Content and APX Activity in Rice Anthers

PA64S anthers were stained with 3,3′-Diaminobenzidine as described in previous studies [90]. ROS was quantified by measuring H_2_O_2_. Briefly, fresh rice anthers were ground to a fine powder using liquid nitrogen and the powder was homogenized with buffer on ice, and centrifugation at 8000× *g* for 10 min at 4 °C. The H_2_O_2_ in the supernatant was quantified as described in the H_2_O_2_ kit (Keming, Suzhou, China). Similarly, APX activity was performed using the plant APX activity assay kit (Keming, Suzhou, China).

### 4.8. UPLC-MS/MS Analysis of Anther Metabolites

Anther samples were freeze dried by vacuum freeze dryer (Scientz-100F). The freeze dried sample was crushed using a mixer mill (MM 400, Retsch) with a zirconia bead for 1.5 min at 30 Hz. A total of 100 mg of lyophilized powder was dissolved with 1.2 mL 70% methanol solution, vortexed for 30 s every 30 min for 6 times in total, the sample was places in a refrigerator at 4 °C overnight. Following centrifugation at 12,000 rpm for 10 min, the extracts were filtrated (SCAA-104, 0.22 μm pore size; ANPEL,Shanghai, China, http://www.anpel.com.cn/, accessed on 11 October 2021) before UPLC-MS/MS analysis. The sample extracts were analyzed using an UPLC-ESI-MS/MS system (UPLC, SHIMADZU Nexera X2, https://www.shimadzu.com.cn/, accessed on 11 October 2021; MS, Applied Biosystems 4500 Q TRAP, https://www.thermofisher.cn/cn/zh/home/brands/applied-biosystems.html, accessed on 11 October 2021).

### 4.9. Data and Figures

The RNA-seq data of the *OsSPL* gene family in 11 various tissues (shoots, leaves, seeds, inflorescence and anthers) were obtained from the public database (Rice Genome Annotation Project, http://rice.plantbiology.msu.edu/index.shtml, accessed on 22 August 2021). Statistical analysis of data and plotting of histograms were performed using Origin 2021. Error bars represented by standard deviations. Asterisks indicated significant differences revealed by the Student’s *t*-test.

## 5. Conclusions

In conclusion, we identified the *SPL* gene family in rice with 19 members. Based on the temperature experimental system for pollen fertility transformations from PTGMS rice lines PA2364S and PA2864S, with different critical temperatures, it was suggested that three *SPL* family members, *OsSPL4*, *OsSPL16* and *OsSPL17*, were involved in regulating male fertility responding to temperature. The WGCNA analysis revealed *CHI* and *APX1* co-expression with *OsSPL17*, and they play an important role in the flavonoid synthesis pathway and ROS clear system, respectively. Flavonoid metabolite analysis revealed that a more active flavonoid biosynthesis process exists in fertile plants, *OsSPL17* negatively regulates *CHI* to increase the biosynthetic process of flavonols and flavonoids, which provide more flavonoid substances for fertile plant fertility formation. In addition, *OsSPL17* negatively regulates *APX1* to affect APX activity, thereby regulating ROS content in the tapetum, controlling the PCD process, and regulating male fertility in rice. The results provide a new insight for further analysis of the *OsSPL* gene regulation of male fertility in PTGMS rice. Further research is needed to understand the molecular mechanism of *OsSPL* in the regulation of male fertility in rice.

## Figures and Tables

**Table 1:** Pollen fertility and seed-setting rate of PA2364S and PA2864S.

Materials	Treatments	Pollen I_2_-KI Dyeing Rate Mean ± SD (%)	Seed Setting Rate Mean ± SD (%)
PA2364S	30 °C	0.00	0.00
	25 °C	0.00	0.00
	21 °C	39.30 ± 0.29 *	34.38 ± 3.29 *
PA2864S	30 °C	0.00	0.00
	25 °C	31.26 ± 0.25 *	30.72 ± 2.69 *
	21 °C	37.35 ± 0.15 *	38.35 ± 1.45 *

Value is expressed as mean ± standard deviation, *n* > 120; asterisks indicate significant differences revealed by Student’s *t*-test at *p* < 0.05 (*).

**Table 2:** Basic information of *Oryza sativa* and *Arabidopsis SPL* genes in different evolutionary groups.

Group	Gene Name	Gene ID	Peptide (aa)	PI	MW (Da)
1	*OsSPL14*	LOC_Os08g39890	417	9.46	42,378.83
	*OsSPL17*	LOC_Os09g31438	323	8.29	33,967.35
	*AtSPL9*	AT2G42200.1	375	8.40	40,846.64
	*AtSPL15*	AT3G57920.1	354	9.11	39,672.42
2	*OsSPL3*	LOC_Os02g04680	282	9.44	30,591.48
	*OsSPL4*	LOC_Os02g07780	251	9.35	28,157.85
	*OsSPL11*	LOC_Os06g45310	343	7.61	37,344.28
	*OsSPL12*	LOC_Os06g49010	475	9.03	50,742.73
	*AtSPL2*	AT5G43270.2	419	8.85	46,860.38
	*AtSPL10*	AT1G27370.1	396	7.94	44,159.12
	*AtSPL11*	AT1G27360.1	393	8.35	43,863.97
3	*OsSPL2*	LOC_Os01g69830	311	9.25	33,328.97
	*OsSPL16*	LOC_Os08g41940	455	7.18	46,578.88
	*OsSPL18*	LOC_Os09g32944	472	7.16	49,646.22
	*OsSPL19*	LOC_Os11g30370	352	8.60	36,657.7
	*AtSPL13*	AT5G50570.1	359	8.03	39,108.44
4	*OsSPL1*	LOC_Os01g18850	862	6.53	95,876.37
	*OsSPL6*	LOC_Os03g61760	969	5.37	105,603.96
	*OsSPL15*	LOC_Os08g40260	1140	7.54	124,430.64
	*AtSPL1*	AT2G47070.1	881	5.55	98,459.93
	*AtSPL12*	AT3G60030.1	927	5.85	104,142.47
5	*OsSPL9*	LOC_Os05g33810	842	5.84	92,018.44
	*AtSPL7*	AT5G18830.3	818	6.13	91,550.37
6	*AtSPL14*	AT1G20980.1	1035	8.71	114,813.64
	*AtSPL16*	AT1G76580.1	1020	8.87	113,394.12
7	*OsSPL5*	LOC_Os02g08070	486	6.34	49,131.79
	*OsSPL10*	LOC_Os06g44860	426	9.15	44,291.94
	*AtSPL8*	AT1G02065.1	333	9.01	36,827.06
8	*OsSPL8*	LOC_Os04g56170	416	7.21	45,029.54
9	*OsSPL13*	LOC_Os07g32170	216	10.19	22,044.27
	*AtSPL3*	AT2G33810.1	131	8.23	15,303.97
	*AtSPL4*	AT1G53160.2	174	9.69	20,119.57
	*AtSPL5*	AT3G15270.1	181	9.82	20,991.54
10	*OsSPL7*	LOC_Os04g46580	360	9.45	37,387.07
	*AtSPL6*	AT1G69170.1	405	7.60	45,952.84

**Table 3:** One to one orthologous relationships between the *SPL* gene members of *Oryza sativa* and *Arabidopsis thaliana*.

OsSPL Gene Name	OsSPL Gene ID	AtSPL Gene Name	AtSPL Gene ID	Ka	Ks	Ka/Ks	Selection Pressure
*OsSPL1*	LOC_Os01g18850	*AtSPL1*	AT2G47070.1	0.5253	2.3771	0.2210	Purifying selection
*OsSPL2*	LOC_Os01g69830	*AtSPL13*	AT5G50570.2	0.6808	2.2521	0.3023	Purifying selection
*OsSPL14*	LOC_Os08g39890	*AtSPL9*	AT2G42200.1	0.6658	2.3289	0.2859	Purifying selection
*OsSPL15*	LOC_Os08g40260	*AtSPL16*	AT1G76580.1	0.4401	2.3330	0.1886	Purifying selection
*OsSPL16*	LOC_Os08g41940	*AtSPL13*	AT5G50570.2	0.6892	2.0674	0.3334	Purifying selection
*OsSPL17*	LOC_Os09g31438	*AtSPL9*	AT2G42200.1	0.7400	1.9131	0.3868	Purifying selection
*OsSPL19*	LOC_Os11g30370	*AtSPL13*	AT5G50570.2	0.7375	2.0284	0.3636	Purifying selection

## Data Availability

Not applicable.

## Supplementary Materials

The following supporting information can be downloaded at: https://www.mdpi.com/article/10.3390/ijms23073744/s1.
