# Supplementary material for: OsSPLs Regulate Male Fertility in Response to Different Temperatures by Flavonoid Biosynthesis and Tapetum PCD in PTGMS Rice
Source: Int J Mol Sci. 2022 Mar 29;23(7):3744. doi: 10.3390/ijms23073744 (PMC8998824; doi:10.3390/ijms23073744)
Supplement: Supplementary file 1 [file ijms-23-03744-s001.zip › ijms-1643568-supplementary.pdf]

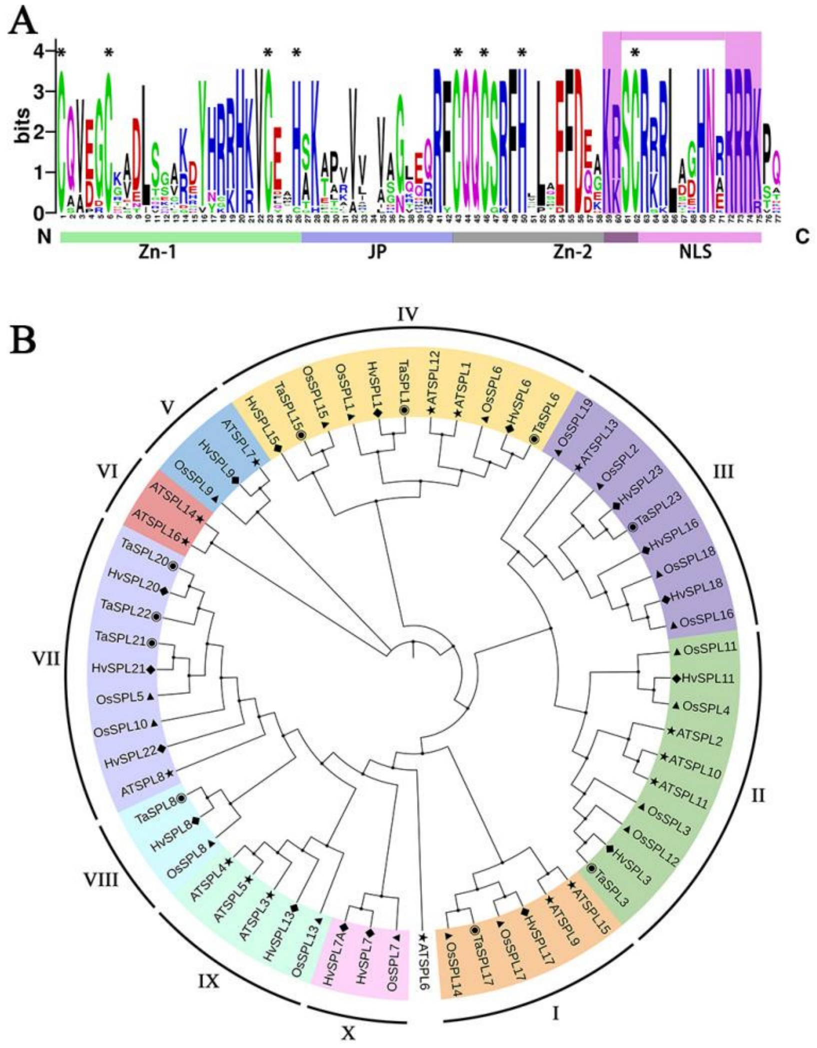

Figure S1. Sequence and phylogenetic analysis of *OsSPL*. (**A**) Visualization of multiple sequence alignment of the *OsSPL* gene family DNA binding domains. (**B**) Phylogenetic analysis of *SPL* gene family in rice, Arabidopsis, Hordeum vulgareand and Triticum aestivum. Zn-1, inc finger 1. Zn-2, zinc finger 2. Jp, joint peptide. NLS, nuclear localization sequence. * indicates the amino acid contained in the structural sequence. As shown Figure S1A, the SBP domain of OsSPL is a conserved amino acid sequence containing four motifs, the zinc finger 1 (Zn-1), zinc finger 2 (Zn-2), the joint peptide (Jp) of the zinc finger and the nuclear localization sequence (NLS). The two zinc fingers structure sequences of Zn-1 and Zn-2 were containing Cys-Cys-Cys-His and Cys-Cys-His-Cys, respectively.

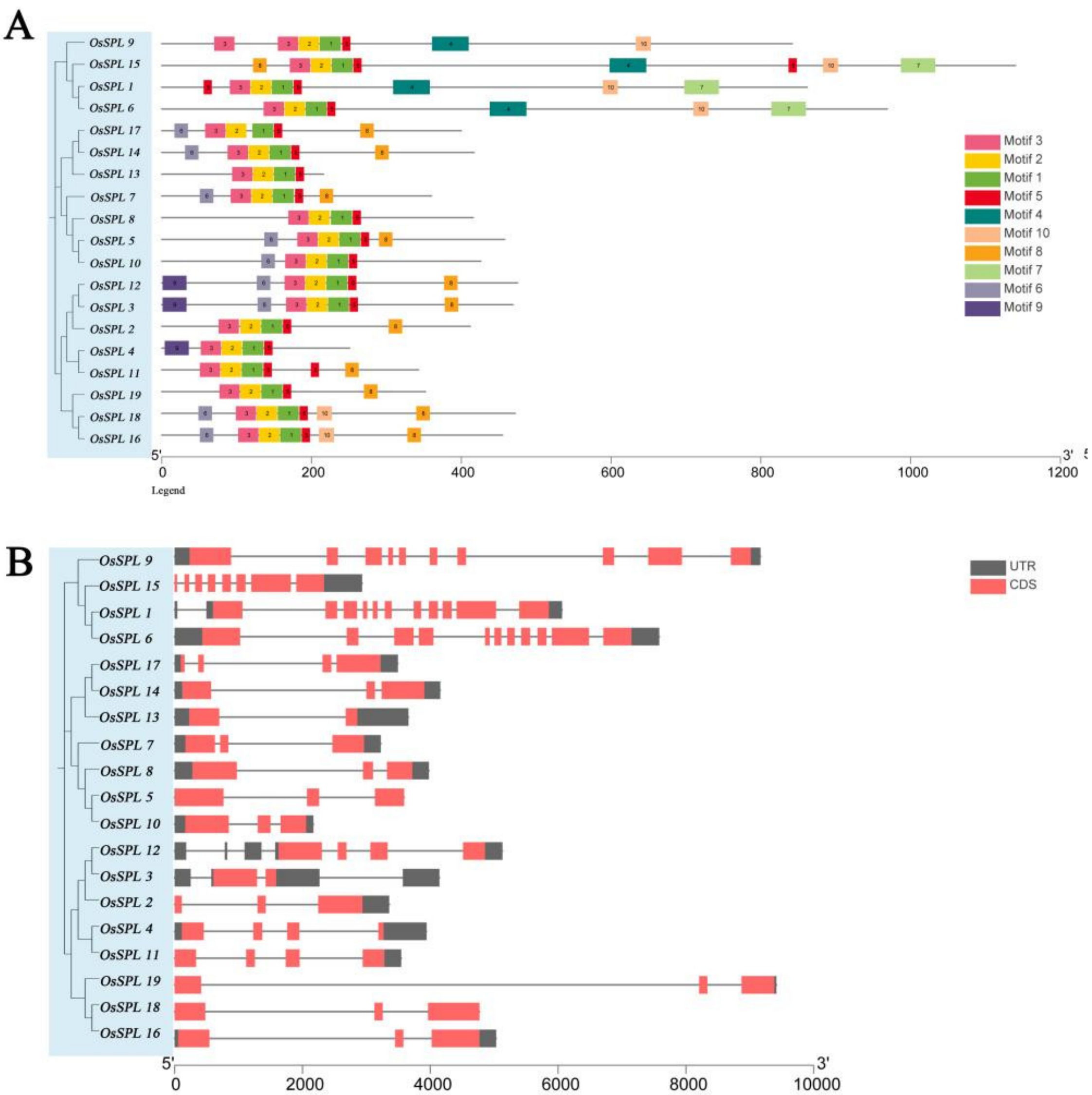

Figure S2. Structure analysis of *OsSPL* genes. (**A**) Conserved motifs DNA structure analysis of *OsSPL* genes based on phylogenetic relationship. (**B**) Motif structure analysis of *OsSPL* proteins based on phylogenetic relationship. Each motif is represented by a colored box.

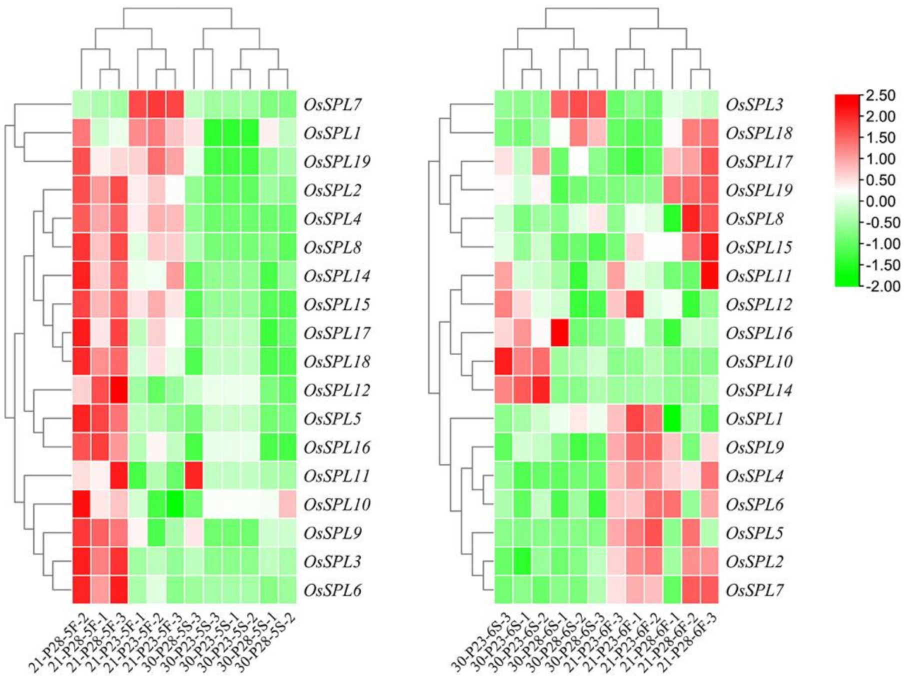

Figure S3. Expression profiles of *OsSPL* genes in 5th and 6th stages of PA2364S and PA2864S. 30/21. Treated at 30°C or 21°C. P23/P28. PA2364S/PA2864S. 5S/5F. 5th stage sterile or fertile plants.

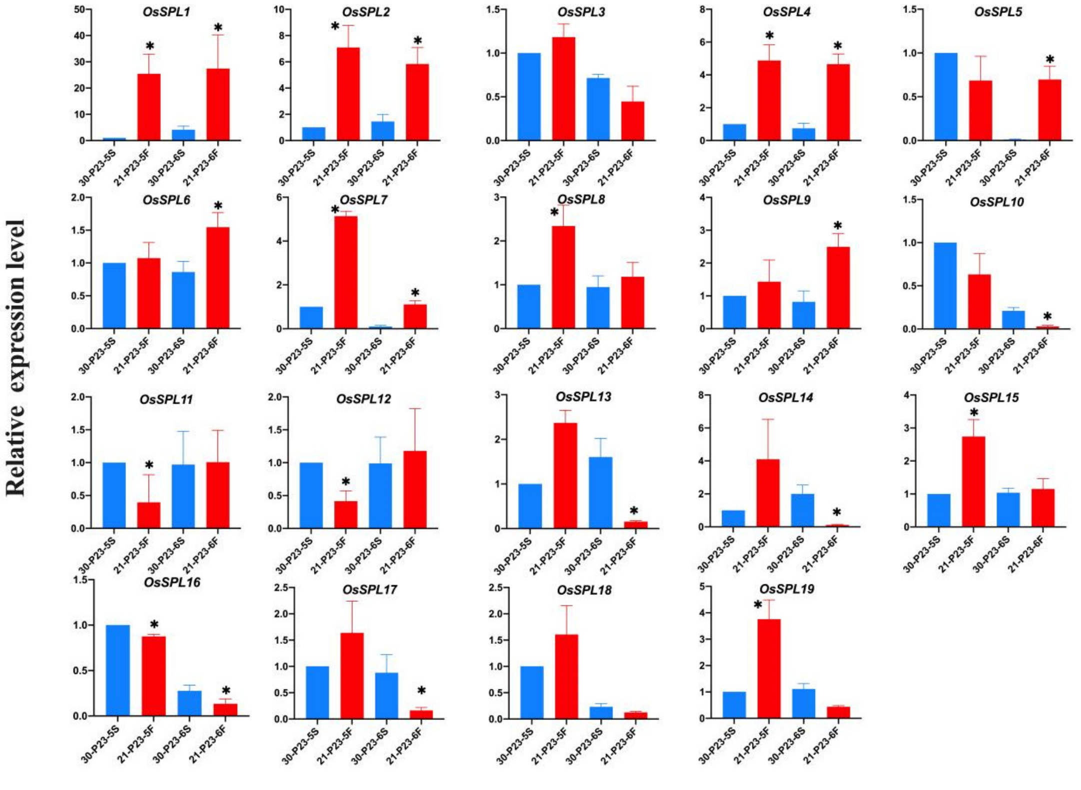

Figure S4. Expression pattern of *OsSPL* gene under different temperature treatments in PA2364S by qPCR. 30/21. Treated at 30°C or 21°C. P23.PA2864S. 5S/5F. 5th stage sterile or fertile plants. For each treatment in the qPCR results we show the average of three replicates. p (*)<0.05.

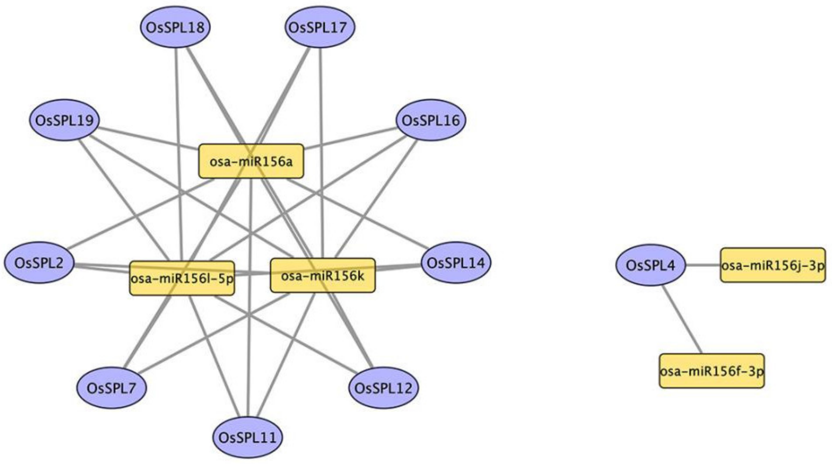

Figure S5. Relationships between miR156 and their 10 targeted *OsSPL* genes.

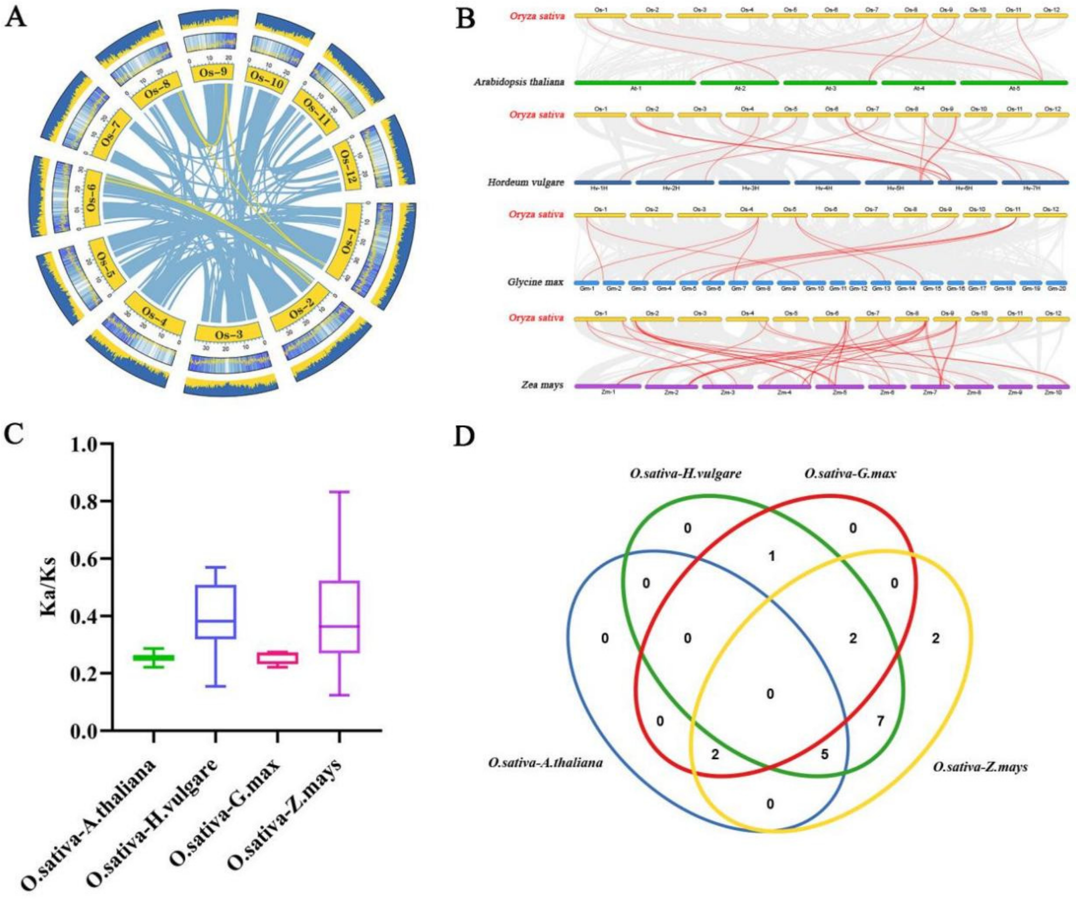

Figure S6. Collinearity analysis of *OsSPL* genes. (**A**) Collinearity analysis of *OsSPL* genes in the rice genome. All the syntenic blocks in the rice genome were depicted by the gray lines, and the red lines linked the duplicated *SPL* gene pairs. Black rectangles were used to indicate chromosomes 1–12. Lines, heat maps and bars inside the rectangles indicate the gene density on the chromosomes. (**B**) Collinearity analysis of *SPL* genes between rice and four other plants. The gray lines indicate gene blocks in rice that are orthologous to the other genomes. The red lines linked the syntenic *SPL* gene pairs. (**C**) The ratio of nonsynonymous to synonymous substitutions (Ka/Ks) of *SP*L genes between rice and four other plant species. (**D**) The Venn diagram of syntenic *SPL* genes between rice and four other plant species.

Table S1. Primer information for mRNAs and miRNA.

Gene Name	Forward Primer	Reverse Primer
miR156a	CCTGACAGAAGAGATTGAGCAC	Reverse Primer
miR156k	TGACAGAAGAGAGAGAGCACA	Reverse Primer
miR156l-5p	GGCGACAGAAGAGAGTGAGCAT	Reverse Primer
OsSPL1	CCTGCAATAGGCAACAAGCA	TCAACCTGGAAAAGAAAACAAGA
OsSPL2	ATACCCCCAAGCCAGCTTAT	TCCTGCTGAACTAGCCCCTA
OsSPL3	TACCCCATCTCCTGGTCTGG	AGACCCGCAACGATGACTTT
OsSPL4	AAAGCTCCACTCAGCCATGT	AGGACACCGAAGAACATCCA
OsSPL5	CAGTGCAGCAGGTTTCATGT	GGTGTAGGAGCTAGCGATGC
OsSPL6	TGACCCTCCAGCATGCAAAT	AGTCTGTGGTGGGCTTTGAG
OsSPL7	CCGCAGCCTTTTTCCCAATC	TAGCGTCGTTACACAGCAGC
OsSPL8	GGGTGCACACATGTCCAAAG	GAGGGAATAAGCACGAGCCA
OsSPL9	CTGGATACTCATGGACCCGC	TCCGAAGAACCACACAGAGC
OsSPL10	CAACATCTTGTCGTGCTCGT	CCATTGTTGTGGCTGTTGTC
OsSPL11	CGACGGAAACCACAGACAGA	TGCATGGTTAAGTGGAGCGT
OsSPL12	AGAAAAGCAGCACCATGGCATA	ATCAAACCGATCCGAGGAGG
OsSPL13	GTGAGTGTGTGTGTTCGTGC	GCCTCTCCAGCATAGCACTG
OsSPL14	GCATCTGTTGGTGAGCATCG	CACTAGAGTGACCATGAGGAG
OsSPL15	CAACAGGCGGCGAAGAAAAA	AGGGATGCTGGGCAGTTTAC
OsSPL16	GTAGAAAGCGACTAGATGGG	GTTTTGATCATCCCTGTCCA
OsSPL17	TCCTTTCATGAGCCAGGCAG	CCACTGGATTGAACCGGACA
OsSPL18	ATGACAAGTCAACAAGTTCTGT	GGGGTCTCCTCGGATTTGAT
OsSPL19	CTTCCCCAGAAGATGGAGCC	TGAAAGAAGAGGGGGCGAAC
CHS	AGTTCAAGAGGATGTGTGACAAG	CATCTTGGCGAGCTGGTAGT
CHI	AAGTTCACGAGGGTGACGAT	AGGCCTCCTTGAACTTGTCC
FNS2	GCGGAAGAAGACAGACACAG	TTGATGTTGTCTCTGGTGAGC
F3H	CATGGACCAGAAGGTTGTCG	CTCCCATTGCTCAGATAGTGA
APX1	TGTCCTTGTCACTCAAACCCATC	GACCAACTTCCCATCCTCTCCTA

Note: Reverse Primer is a special primer in Tiangen kit.
